# Sleep quality and influencing factors and correlation with T-lymphocyte subpopulation counts in patients with pulmonary tuberculosis: a cross-sectional study

**DOI:** 10.1186/s12879-022-07946-7

**Published:** 2022-12-22

**Authors:** Hailin Zhang, Ao Li, Youli Jiang, Wenqiu Chen, Jin Wang, Peize Zhang, Guofang Deng, Weiyu Wang, Jingfang Chen, Yi Lin

**Affiliations:** 1 Department of Pulmonary Medicine and Tuberculosis, The Third People’s Hospital of Shenzhen, Shenzhen, People’s Republic of China; 2 Hengyang Medical School, School of Nursing, University of South China, Hengyang, People’s Republic of China

**Keywords:** Pulmonary tuberculosis, Sleep quality, T-lymphocyte subpopulation count, PSQI, Influencing factors

## Abstract

**Background:**

Patients diagnosed with pulmonary tuberculosis (TB) have poor sleep quality due to multiple factors. We aimed to assess the sleep status and related factors of TB patients in Shenzhen, China.

**Methods:**

A questionnaire survey was conducted on 461 TB patients hospitalized at Shenzhen Third People’s Hospital from March 2021 to January 2022, and sleep quality was assessed using the Pittsburgh sleep quality index (PSQI).

**Results:**

A total of 459 valid questionnaires were collected, and 238 of the 459 TB patients had general or poor sleep quality (PSQI > 5). Patients’ gender, marriage, nutritional screening score, family atmosphere, fear of discrimination, fear of interactions, and the impact of the disease on their work life had significant effects on sleep quality (*P* < 0.05); PSQI scores of TB patients were negatively correlated with lymphocyte counts (*r* =  − 0.296, *P* < 0.01), T-lymphocyte counts (*r* =  − 0.293, *P* < 0.01), helper T lymphocyte counts (*r* =  − 0.283, *P* < 0.01), killer T lymphocyte counts (*r* =  − 0.182, *P* < 0.05), and were positively correlated with depression scores (*r* = 0.424, *P* < 0.01). Multivariable logistic regression analysis showed that male (OR = 1.64,95% CI 1.11–2.42, *P* < 0.05), unmarried (OR = 1.57, 95% CI 1.02–2.42, *P* < 0.05), NRS score grade 3(OR = 5.35, 95% CI 2.08–15.73, *P* < 0.01), general family atmosphere (OR = 2.23, 95% CI 1.07–4.93, *P* < 0.05), and the disease affecting work (OR = 1.66, 95% CI 1.11–2.50, *P* < 0.05) were factors influencing poor sleep quality.

**Conclusion:**

Most TB patients had varying degrees of sleep disturbance, which may be affected by their gender, marriage, family atmosphere, nutritional status, the effect of the disease on work life, and, depression, as well as lower absolute T-lymphocyte subpopulation counts. Appropriate interventions should be implemented to improve their sleep quality, when treating or caring for such patients.

**Supplementary Information:**

The online version contains supplementary material available at 10.1186/s12879-022-07946-7.

## Introduction

Pulmonary Tuberculosis (TB) is a respiratory infectious disease caused by *Mycobacterium tuberculosis* infection, which seriously affects people’s health and is mainly transmitted through the respiratory tract. According to the 2020 World Health Organization Global Tuberculosis Report [1], about 10 million people worldwide were infected with TB in 2019, and China ranked third, with about 840,000 newly diagnosed patients.

TB is a chronic wasting disease, and a high proportion of TB patients experienced malnutrition and weakness [2]. In addition, TB treatment is costly, and patients must withdraw from work to receive treatment sometimes, which puts an extra financial strain on their families [3]. Moreover, there is currently a lack of public awareness about TB, and the condition has long been stigmatized worldwide [4]. Within such a social context, TB patients are put under a heavy psychological burden for an extended amount of time, and their sleep quality can be negatively affected.

In current Chinese society, most people are prejudiced against TB patients, fearing that contact with them will lead to transmission of the disease, so TB patients are often forced to conceal their condition and inevitably have low self-esteem [5]. Combined with uncertainty and concern about recovery from the disease, patients may experience a poorer psychological state and are at a much higher risk of disrupted sleep. The relationship between TB and sleep deprivation has been confirmed in studies [6]. In a study in India, poor sleep quality was reported to be approximately three times higher in TB patients than in the normal population (17% and 6.2%, respectively) [7], so it is essential to assess the quality of sleep in TB patients. Currently, the sleep status of Chinese TB patients remains largely unassessed.

Sleep quality is often overlooked in clinical settings, with treatment and care of disease receiving more attention from medical professionals. However, studies have shown that sleep quality correlates with health issue, and that high quality sleep reduces the risk of medical problem and even promotes improvement in patients [8–10]. Adequate sleep can lay the foundation for a day of work and study, while people with insufficient and poor sleep quality may be lethargic, which can affect the patient’s ability to recover from illness and may even cause the patient to lose confidence in the medical care they are receiving. Huang et al. showed that poor sleep may be associated with poor disease prognosis [11]. Thus, the concern for study into sleep disorders in patients with TB is not only to improve their quality of life, but is also directly connected to their long-term prognosis.

The immune mechanism of TB is complex, and the body, after invasion by *Mycobacterium tuberculosis*, generates a series of cellular immunities that allows the antigen to reach T cells and produce a specific response. T cells play an important role in the immune response to TB, and the measurement of peripheral blood T cells can reflect the immune status of the body [12]. Analysis of T-cell subpopulation has been widely used in clinical diagnosis and screening of TB, and is of great value in assessing the extent of the disease, TB activity, treatment efficacy, and prognosis. However, whether and how T-cell subtype counts correlate with sleep quality in TB patients is still unclear.

The purpose of this observational study was primarily to assess the sleep of patients with TB and the factors that may affect their sleep quality. We hypothesized that poor sleep quality in TB patients may be related to the psychological distress experienced after diagnosis and while undergoing treatment. We explored the extent to which different demographic characteristics predict poor sleep quality in TB patients and the correlation between indicators of T-lymphocyte subsets and sleep quality in TB patients.

## Methods

### Design and samples

Shenzhen Third People's Hospital, a tertiary care hospital that serves as a national clinical research center for infectious diseases, admits patients with difficult-to-diagnose as well as refractory TB from across China. The hospital has 2,608 beds and undertakes major epidemic prevention and control tasks in Shenzhen and surrounding areas. A total of 461 patients admitted with TB from March 1, 2021, to January 31, 2022, were selected for this study using a convenience sampling method. Inclusion criteria were patients aged ≥ 14 years diagnosed with either bacteriologically-confirmed or clinically diagnosed TB; clear consciousness and smooth communication; and voluntary participation in the study. Exclusion criteria included patients who were critically ill, unable to cooperate with the investigation, suffered with psychiatric disorders, had epilepsy or, other central nervous disorders, or provided incomplete information.

The purpose of this cross-sectional study was to assess the sleep quality of Chinese patients with TB. The probability of poor sleep quality in patients with TB was expected to be 17% based on the literature [5], with a tolerance error of 0.03, requiring a two-sided test of 0.05. According to the formula $n=\frac{{{Z}_{\alpha }}^{2}*{\sigma }^{2}}{{\delta }^{2}}$, the sample size of n = 339 cases was required, and accounting for 5% invalid questionnaires, at least 357 cases needed to be included as study subjects.

### Ethical consideration

The study protocol was approved by the Ethics Review Committee of the Shenzhen Third People's Hospital (No. 2020-019-03). The purpose of the study was explained in detail to the patients at the time the questionnaires were distributed to them. All patients participating in the study, or their guardians, signed informed-consent forms, and the information collected was used for research purposes only.

### Measurement

A pre-tested, semi-structured questionnaire consisting of four parts was administered to study participants by trained researchers ‘An additional file shows this in more detail (see Additional file 1)’. The first part was a patient admission questionnaire, which included health insurance details, domestic circumstances, family atmosphere, concerns about the diagnosis of TB, and the impact of TB on themselves, followed by a nutrition risk screening (NRS) 2002 score, a Self-Rating Depression Scale (SDS), and the Pittsburgh Sleep Quality Index (PSQI). In this study, little communication or even indifference between family members was defined as “a general family atmosphere”, which was self-evaluated by the patients after the data collector explained it to them. The NRS 2002 is a tool proposed by Professor Kondrup and recommended by the European Dietetic Association in 2002 as the tool of choice for nutritional risk screening in inpatients [13]. The SDS scale was used in this study to assess the level of psychological depression in TB patients. In the previous studies, the sensitivity and specificity of the SDS scale used to assess depression levels were 79.2% and 72.2%, respectively, and the positive and negative predictive values were 12.5% and 98.6%, respectively [14]. The Cronbach alpha for the Chinese version of the PSQI was 0.71 and the intraclass correlation coefficient was 0.90. The optimal threshold value for detecting poor sleep quality was five, with a sensitivity of 0.81 and specificity of 0.70 [15]. Therefore, this study used a PSQI score of five as the determining line between good and poor sleep quality.

A T-lymphocyte subpopulation assay was performed in the patients with TB, operated by a technician specializing in laboratory science, using a BD FACSCanto II flow cytometer. The principle of the test is a four-color direct immunofluorescence method, according to the different expression of CD molecules on the lymphocyte membrane, fluorescein-labeled monoclonal antibodies of various kinds are added to whole blood to bind with antigens on the leukocyte membrane and made into a suspension after hemolysis. The percentages of T-lymphocyte subpopulations, including T-lymphocytes, helper T-lymphocytes, killer T-lymphocytes, and CD4 + /CD8 +, as well as their absolute counts, were analyzed on flow cytometer computer software. The results were uploaded to the hospital information system (HIS) after all the assays were completed. This test was done within 24 h of the patients’ admissions.

### Data collection

The method of completing the questionnaire was explained to the participants, and patient immunological indicators, as well as hospitalization information, were retrieved from the HIS. A total of 461 questionnaires were distributed in this study, and 459 were returned, giving a questionnaire return rate of 99.6%. All returned questionnaires were valid and were included in the analysis.

### Data management

Data entry of the collected questionnaires was done by Wenqiu Chen and Hailin Zhang separately, and both data were checked for accuracy upon completion. The study data were kept in a combination of paper backups and electronic copies. The preservation of all research data is performed by Yi Lin.

### Data analysis

Statistical analysis of the data was performed using R version 4.1.3. Demographic characteristics were summarized using descriptive statistics. Quantitative data were presented as the mean ± standard deviation or median and quartiles (upper and lower quartiles). Continuous variables were tested for Shapiro–Wilk normality, and differences between groups were assessed using the independent samples t-test for data normally distributed or nonparametric tests (Mann–Whitney test) for data not normally distributed. The categorical variables were expressed as absolute numbers and percentages and were compared using chi-square tests. Pearson linear correlation was used to analyze the correlation between immunological indicators and other indicators, and subgroup analysis was performed for immunological indicators with significant correlations ‘An additional figure file shows this in more detail (see Additional file 2)’. Multivariate logistic regression equations were constructed using logistic stepwise forward (LR) method, and variables with a *P* value of < 0.1 in the univariate analysis were introduced into the multivariate model. The results were expressed as adjusted odds ratios (ORs) with 95% confidence intervals (95% CIs). A two-sided test of P < 0.05 was considered statistically significant.

## Results

Of the 459 patients, 271 were male and 188 were female, with an average age of 42 years. Among them, 162 were unemployed, and 76 were self-financed, i.e., without any health insurance subsidy. The percentage of TB patients living alone was 22.9%. In the survey of sleep quality based on the PSQI scale, 51.9% of TB patients self-reported poor sleep quality (PSQI > 5), as shown in Table 1.

**Table 1:** General admission information of participants (N = 459)

Variable	Total	Poor Sleep (n = 238)	Good Sleep(n = 221)	*P* value
		n (%)	n (%)	
Age (years)				
< 18	5 (1)	3 (1.3)	2 (0.9)	0.589
19–40	240 (52.3)	119 (50)	121 (54.7)	
41–60	155 (33.8)	85 (35.7)	70 (31.7)	
> 60	59 (12.9)	31 (13)	28 (12.7)	
Gender				
Male	271 (59)	155 (65.1)	116 (52.5)	0.006
Female	188 (41)	83 (34.9)	105 (47.5)	
Education				
Junior High School and below	226 (49.2)	118 (49.6)	108 (48.9)	0.879
High School and above	233 (50.8)	120 (50.4)	113 (51.1)	
Marriage				
Unmarried	135 (29.4)	82 (34.6)	53 (24)	0.014
Married	324 (70.6)	156 (65.5)	168 (76)	
Employment status				
Unemployed	162 (35.3)	84 (35.3)	78 (35.3)	1.000
Employed	297 (64.7)	154 (64.7)	143 (64.7)	
Medical insurance				
Yes	383 (83.4)	194 (81.5)	189 (85.5)	0.248
No	76 (16.6)	44 (18.5)	32 (14.5)	
Living alone				
Yes	105 (22.9)	63 (26.5)	42 (19)	0.057
No	354 (77.1)	175 (73.5)	179 (81)	
Operation history				
Yes	34 (7.4)	20 (8.4)	14 (6.3)	0.398
No	425 (92.6)	218 (91.6)	207 (93.7)	
Family atmosphere				
Good	424 (92.4)	214 (89.9)	210 (95)	0.039
General	35 (7.6)	24 (10.1)	11 (5)	
Disease course				
1 month	297 (64.7)	156 (65.5)	141 (63.8)	0.769
1 month ~ 1 year	107 (23.3)	56 (23.5)	51 (23.1)	
> 1 year	55 (12)	26 (11)	29 (13.1)	
In-ward frequency				
First	397 (86.5)	204 (85.7)	193 (87.3)	0.613
Again	62 (13.5)	34 (14.3)	28 (12.7)	
NRS2002 score				
0	116 (25.3)	51 (21.4)	65 (29.4)	0.012
1	228 (49.7)	114 (47.9)	114 (51.6)	
2	49 (10.7)	28 (11.8)	21 (9.5)	
3	28 (6.1)	22 (9.2)	6 (2.7)	
4	38 (8.2)	23 (9.7)	15 (6.8)	
Depression score		50.54** ± **10.53	42.65** ± **9.75	< 0.001

Patients were divided into two groups according to whether the PSQI score was greater than five. Twenty-one variables such as gender, age, education, employment status, and medical insurance were included. Patients who were male or unmarried were more likely to have a poor sleep quality (PSQI > 5). In addition, a general family atmosphere or the patient’s nutritional status was associated with sleep quality (Table 1). There were no significant differences between the two groups with regards to age, education, employment status, presence of medical insurance, or whether they were living alone.

In terms of psychological issues, patients who worried about discrimination, interpersonal communication, or if TB has affected their work were more likely to have a poor sleep quality (*P* < 0.05). Other factors, such as fear of transmission, fever, cough, chest pain, dyspnea, hemoptysis, surgical history, duration of illness, and number of hospitalizations had no statistically significant effect on patients' sleep quality (*P* > 0.05), as shown in Table 2

**Table 2:** Table of psychological concerns and physical signs on admission in patients with TB

Variable	n (%)	Poor sleep (n = 238)	Good sleep (n = 221)	*P* value
		n (%)	n (%)	
Worry about discrimination				
Yes	260 (56.7)	146 (61.3)	114 (51.6)	0.035
No	199 (43.3)	92 (38.7)	107 (48.4)	
Worry about interpersonal communication				
Yes	304 (66.2)	168 (70.6)	136 (61.5)	0.041
No	155 (33.8)	70 (29.4)	85 (38.5)	
Worry about transmission				
Yes	382 (83.2)	197 (82.8)	185 (83.7)	0.788
No	77 (16.8)	41 (17.2)	36 (16.3)	
Affected their work				
Yes	174 (37.9)	104 (43.7)	70 (31.7)	0.008
No	285 (60.1)	134 (56.3)	151 (68.3)	
Pectoralgia				
Yes	37 (8.1)	24 (10.1)	13 (5.9)	0.098
No	422 (91.9)	214 (89.9)	208 (94.1)	
Cough				
Yes	280 (61.0)	153 (64.3)	127 (57.5)	0.134
No	179 (39.0)	85 (35.7)	94 (42.5)	
Fever				
Yes	42 (9.2)	27 (11.3)	15 (6.8)	0.091
No	417 (90.8)	211 (88.7)	206 (93.2)	
Dyspnea				
Yes	24 (5.2)	17 (7.1)	7 (3.2)	0.056
No	435 (94.8)	221 (92.9)	214 (96.8)	
Hemoptysis				
Yes	47 (10.2)	25 (10.5)	22 (10.0)	0.846
No	412 (89.8)	213 (89.5)	199 (90.0)	

**Fig. 1:**
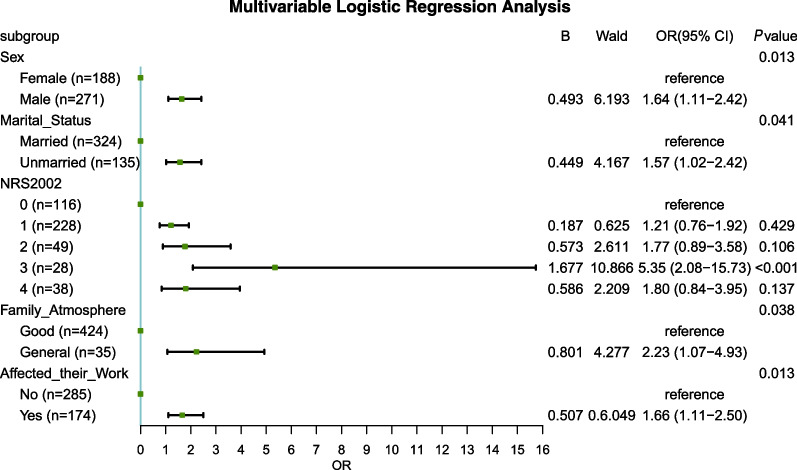
Multivariable logistic regression analysis

In the regression model analysis, variables with a *P* value of < 0.1 in the univariate analysis were introduced into the multivariate model, including gender, marriage status, whether they were living alone, NRS 2002 score, family atmosphere, fear of interpersonal communication, fear of discrimination, dyspnea, and chest pain. Multivariate logistic regression equations were constructed using the LR method, and five variables were finally included in the regression model. The results showed that patients with a high NRS score (grade 3, OR = 5.35, 95%CI 2.08–15.73, *P* < 0.01), general family atmosphere (OR = 2.23, 95% CI 1.07–4.93, *P* < 0.05), who were male (OR = 1.64, 95 CI 1.11–2.42, *P* < 0.05), unmarried (OR = 1.57, 95%CI 1.02–2.42, *P* < 0.05) and patients with a diagnosis of tuberculosis that affected their work (OR = 1.66, 95% CI 1.11–2.50, *P* < 0.05) were associated with a higher risk of poor sleep quality. The results of which are shown in Fig. 1.

In total, 249 of the 459 patients were tested for T-cell subsets, of which 127 patients with poor sleep had a lymphocyte count of 1.18 (0.86, 1.46)/μl, T-lymphocyte counts 737 (533,980)/μl, helper T-lymphocyte counts 424 (287,548)/μl, and killer T-lymphocyte count 273 (185,407)/μl, the differences in these indicators were statistically significant (*P* < 0.01) compared with patients with good sleep quality. The difference between the groups of T cells, CD4 + /CD8 +, helper T cells, and killer T cells was not statistically significant (P > 0.05), as shown in Table 3.

**Table 3:** T-lymphocyte subpopulation assay and depression score in both groups

Variable	Poor sleep (n = 127)	Good sleep (n = 122)	*P* value
	Median (IQR)	Median (IQR)	
Lymphocyte cell counts (U/L)	1.18 (0.86–1.46)	1.29 (1.02–1.63)	0.003
T cell (%)	67.4 (59.8–73.9)	68.8 (58.68–74.1)	0.690
T cell counts (/μl)	737 (533–980)	850 (642.5–1101)	0.001
CD4 + /CD8 +	1.46 (1.09–1.90)	1.42 (0.99–1.95)	0.680
Helper T cell (%)	36.90 (30–842.2)	36.75 (31.2–43)	0.771
Helper T cell counts (/μl)	424 (287–548)	491 (362.75–596)	0.001
Killer T cell (%)	25.7 (19.7–31.3)	25.6 (19.78–32.15)	0.913
Killer T cell counts (/μl)	273 (185–407)	327 (237–437)	0.005

**Fig. 2:**
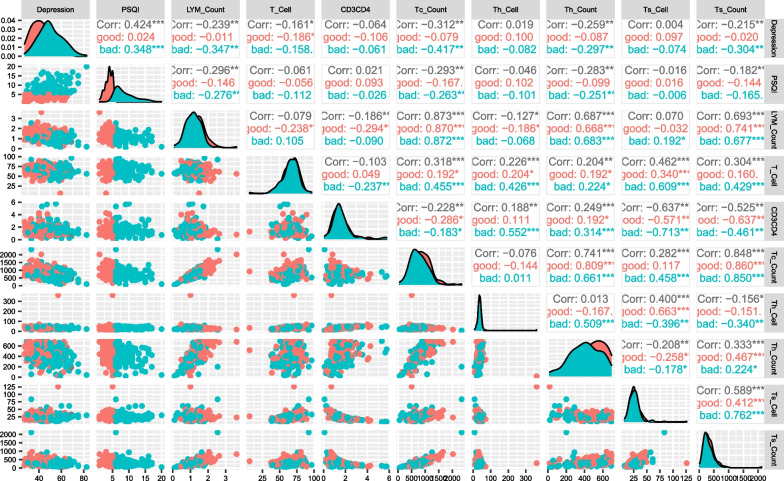
Pearson correlation analysis

Immunological indicators, as well as depression scores and PSQI scores of the included patients, were analyzed by Pearson linear correlation, and the results showed that the PSQI scores for the TB patients were negatively correlated with lymphocyte counts (r =  − 0.296, *P* < 0.01), T lymphocyte counts (r =  − 0.293, *P* < 0.01), helper T lymphocyte counts (r =  − 0.283, *P* < 0.01), and killer T lymphocyte count (r =  − 0.182, *P* < 0.05), and there was a positively correlation with the depression score (r = 0.424, *P* < 0.01). The results are shown in Fig. 2.

## Discussion

In this cross-sectional study in a tertiary care hospital in Shenzhen, China, more than half (51.9%) of TB patients reported poor sleep quality. In a multifactorial logistic regression analysis, gender, marriage, whether TB diagnosis has affected their work, nutritional status, and family atmosphere were identified as the main factors influencing sleep quality among TB patients. Importantly, we found negative correlations between PSQI scores and absolute counts of lymphocyte subsets, which had not been previously reported. Our findings have important implications for the development of appropriate programs to improve the sleep quality of patients with TB.

The findings showed a higher risk of poor sleep quality in men compared to women with TB, contrary to our hypothesis, and further studies are needed to explore the reasons for this. In a previous study, the risk of poor sleep quality was approximately 1.5 times higher in women than in men [16]. However, our findings found that the risk of TB-related poor sleep quality in this study is higher in men than in women. Reasons for such a sex difference are still unknown, which needs further investigation. In addition, unmarried TB patients are at increased risk of poor sleep quality compared to married patients, which is within our expectations. Hatherall et al. (2019) conducted interviews with TB patients in South Asia based on rooting theory, where TB caused role disruption for the females, leading to a loss of confidence in marital prospects among TB patients [17]. Unmarried patients with a diagnosis of TB may experience a strong negative impact on sleep quality compared to married patients under the dual effects of a lack of support from a loved one and a potentially reduced prospect of marriage.

TB patients are chronically depleted and have a poor nutritional status for a prolonged period after the disease. Our results suggest that nutritional scores are an independent influence on sleep quality, which is similar to the findings from a cross-sectional study conducted at the University of Perugia, Italy, which showed a correlation between malnutrition and sleep disturbance [18]. Although the mechanism is currently unclear, the relationship between nutrition and sleep is apparent, and it may be that diet affects sleep quality through melatonin and its biosynthesis from tryptophan [19]. The current results of research indicates that when adequate sleep and nutrition, which are basic physiological needs, are not achieved, the situation of TB patients can deteriorate further.

Although the cure rate for TB has increased, its infectiousness has led to a significant increase in discrimination against TB patients. In a study conducted in Shandong, China, examining psychological distress in TB patients, 58.6% of TB patients had a Kessler 10 score of 16 or higher, indicating moderate to severe psychological distress [20]. TB patients generally have low self-esteem, and there is a correlation between this psychological state and depressive tendencies [21]. After stepwise regression analysis for all variables was included, the presence or absence of interference with work was correlated with poorer sleep quality (*P* < 0.05). This can easily be explained by the fact that, despite TB being largely curable, TB patients are not treated normally in their daily lives, and sometimes other people, even family members, seek to avoid them. TB stigma refers to a marker of shame that sets a person apart and brings with it feeling of shame or blame. A study by Lee et al. (2017) noted a positive correlation between higher levels of disease stigma and depression in TB patients [22]. Patients often have to endure strange reactions from others while battling the disease. One of the consequences of the long–term stigmatization of TB is that many, patients had to temporarily withdraw from work after diagnosis [23]. The medical treatment of, the disease increases family expenses, which is coupled with a loss in financial resources, and patients face a double dilemma, resulting in a subsequent decrease in sleep quality.

Patients’ depressive psychology was identified as one of the factors affecting their sleep quality. Indeed, difficulty achieving sleep is a major symptom of depression, and people suffering from this psychological condition often have trouble sleeping at night. Several, studies have confirmed this interaction between depression and sleep quality [24, 25]. Depression and sleep disorders frequently co-occur, and the introduction of polysomnography into psychiatric studies confirms continued disturbed sleep patterns in depressed patients [26]. This study’s findings of influencing factors corroborate previous studies. Targeted psychological interventions for patients with TB should be recommended to improve patient compliance with treatment and improve their quality of life; these interventions also have an important impact on the treatment of TB [27].

We analyzed the correlation between lymphocyte subpopulation and sleep quality in patients with TB. The results showed that PSQI scores of TB patients were negatively correlated with the lymphocyte counts, T-lymphocyte counts and helper T lymphocytes counts, which had not been reported previously. Several studies have explored the relationship between CD4 + cell counts and the incidence of TB, and in the study by Geremew et al. [28], the incidence of TB among HIV patients with baseline measured CD4 + cell counts < and > 200 cell/mm^3^ was 28.86% (95%CI 18.73–38.98%) and 13.7% (95%CI 1.41–25.98%), indicating the predictive role of CD4 + cell counts for TB. In another study, compared to healthy control populations and latent TB patients, active TB patients had reduced numbers of CD4 + T and CD8 + T cells [29]. Also, the previous studies showed that T lymphocytes and its subsets negatively correlated with disease severity of TB and the extent of pulmonary lesions [30, 31], which may partially explain the association between decrease of lymphocyte counts and poor sleep quality of TB patients. However, confirmation of these findings will require further investigation in longitudinal cohorts with a larger sample size.

Our study was limited by its cross-sectional design, single-center site, and unavoidable selection bias. Caution must be used when generalizing the results of this study to a different population. In addition, the factors influencing poor sleep quality in TB patients such as family atmosphere, emotional worries, and depression are not easily measured. Interviews with patients are needed in future studies to obtain explanations and supplemental information in the form of qualitative data, which can be relevant for the development of improved measures for helping TB patients.

## Conclusion

This study demonstrated, for the first time, that the sleep quality of Chinese patients with TB was negatively correlated with the absolute counts of lymphocytes, T-cell counts, and helper T-cell counts, although the underlying mechanism is unclear. In addition, male, unmarried, the disease affecting work, poor nutritional status, general family atmosphere, and depression were identified as independent risk factors for poorer sleep quality in TB patients, indicating that special attention and psychological interventions may be required for such TB patients.

## Supplementary Information

**Additional file 1.** Patient Admission Survey Assessment Form: A pre-tested admission questionnaire for TB patients, including a survey of patients’ psychological concerns, a SDS scale, a PSQI scale and a NRS 2002 scale.

**Additional file 2. **A subgroup analysis of immunological indicators was performed for the two groups of patients, and all variables with statistically significant differences were included in the figure.

## Data Availability

The raw data supporting the conclusions of this article will be made available by the corresponding author Yi Lin (514195263@qq.com), without undue reservation.

## Abbreviations

TB: Tuberculosis
NRS: Nutrition risk screening
SDS: Self-Rating depression scale
PSQI: Pittsburgh sleepiness quantifier inventory
HIS: Hospital information system
IQR: Interquartile range
LR: Logistic stepwise forward
OR: Odds ratios
CI: Confidence interval
