# Supplementary material for: Sleep quality and influencing factors and correlation with T-lymphocyte subpopulation counts in patients with pulmonary tuberculosis: a cross-sectional study
Source: BMC Infect Dis. 2022 Dec 22;22:956. doi: 10.1186/s12879-022-07946-7 (PMC9773432; doi:10.1186/s12879-022-07946-7)
Supplement: Supplementary file 1 — Additional file 1. Patient Admission Survey Assessment Form: A pre-tested admission questionnaire for TB patients, including a survey of patients’ psychological concerns, a SDS scale, a PSQI scale and a NRS 2002 scale. [file 12879_2022_7946_MOESM1_ESM.pdf]

Patient Admission Survey Assessment Form

Bed No_____ Name_____ Inpatient number_____ Date of filling form____

1. Payment method of hospitalization expenses: ☐oral medical insurance

   ☐at one's own expense ☐medical insurance in different places.

2. Cohabitant: ☐parents ☐spouse ☐Spouses and children ☐children

   ☐brothers and sisters ☐other ☐No one

3. Family Atmosphere: ☐Well ☐General ☐Bad ☐Other

4. Are there any tuberculosis patients in your family? ☐Yes ☐No

5. Are you worried about the spread of tuberculosis to your family, relatives and friends? ☐Yes ☐No

6. Are you worried about the further development of the disease? ☐Yes ☐No

7. Are you worried that your illness will affect your marriage? ☐Yes ☐No

8. Are you worried that your illness will affect your work? ☐Yes ☐No

9. Are you worried that your illness will affect your relationship with others?

   ☐Yes ☐No

10. Are you worried that your illness will be discriminated against by others?

    ☐Yes ☐No

11. Are you worried that the cost of treatment will increase your financial burden?

    ☐Yes ☐No

12. Are you worried that carrying tuberculosis will affect your future? ☐Yes ☐No

13. In what areas are you affected or have you been affected by tuberculosis?

☐Family ☐Marriage ☐Work ☐Interpersonal

☐Communication ☐Economic Burden ☐Future

□Other: What other aspects of your influence are not mentioned above, and hope that our medical staff can help solve them? Please fill in: ___________________________

Table 2 Please read each item carefully, understand the meaning of the topic, and then rate the following 20 items according to your actual situation in the past week: ① Rarely; ② Sometimes; ③ Often; ④ Continuous.

1. I feel depressed and depressed. ① Rarely ② Sometimes ③ Often ④ Continuous
2. I feel the best mood in the morning ① Rarely ② Sometimes ③ Often ④ Continuous
3. I used to cry or want to cry. ① Rarely ② Sometimes ③ Often ④ Continuous
4. I don't sleep well at night. ① Rarely ② Sometimes ③ Often ④ Continuous
5. I eat as much as usual. ① Rarely ② Sometimes ③ Often ④ Continuous
6. My sexual function is normal. ① Rarely ② Sometimes ③ Often ④ Continuous
7. I feel I have lost weight. ① Rarely ② Sometimes ③ Often ④ Continuous
8. I worry about constipation. ① Rarely ② Sometimes ③ Often

④ Continuous

9. My heart beats faster than usual. ① Rarely ② Sometimes ③ Often ④ Continuous

10 I feel tired for no reason. ① Rarely ② Sometimes ③ Often ④ Continuous

11. My mind is as clear as ever. ① Rarely ② Sometimes ③ Often ④ Continuous

12. I don't find it difficult to do things as usual. ① Rarely ② Sometimes ③ Often ④ Continuous

13. I am restless and it is difficult for me to keep calm. ① Rarely ② Sometimes ③ Often ④ Continuous

14. I feel hopeful about the future. ① Rarely ② Sometimes ③ Often ④ Continuous

15. I get angry more easily than usual. ① Rarely ② Sometimes ③ Often ④ Continuous

16. I think it's easy to decide what to do. ① Rarely ② Sometimes ③ Often ④ Continuous

17. I feel I am useful and indispensable. ① Rarely ② Sometimes ③ Often ④ Continuous

18. My life is very meaningful. ① Rarely ② Sometimes ③ Often ④ Continuous

19. If I were dead, others would be better off. ① Rarely ② Sometimes ③ Often ④ Continuous

20. I still like what I usually like. ① Rarely ② Sometimes ③ Often ④ Continuous

Table3 Sleep in the last month

E1 what time do you usually go to bed?	__ __hour__ __minutes
E2 how long does it usually take you to fall asleep every night?	__ __hour__ __minutes
E3 what time do you usually get up every morning?	__ __hour__ __minutes
E4 how much time do you actually sleep every night? (not equal to bed rest time)	__ __hour__ __minutes
Do you often have poor sleep because of the following problems:	
E5 difficulty falling asleep (unable to fall asleep within 30 minutes)?	0□No 1□< 1 time/week 2□1~2 times/week 3□ ⩾3 times/week
E6 Is it easy to wake up or wake up early in sleep?	0□No 1□< 1 time/week 2□1~2 times/week 3□ ⩾3 times/week
E7 Do you get up and go to the bathroom at night?	0□No 1□< 1 time/week 2□1~2 times/week 3□ ⩾3 times/week
E8 Uncomfortable breathing?	0□No 1□< 1 time/week 2□1~2

	times/week 3□ ⩾3 times/week
E9 Coughing or snoring loudly?	0□No 1□< 1 time/week 2□1~2 times/week 3□ ⩾3 times/week
E10 Feel cold?	0□No 1□< 1 time/week 2□1~2 times/week 3□ ⩾3 times/week
E11 Feel too hot?	0□No 1□< 1 time/week 2□1~2 times/week 3□ ⩾3 times/week
E12 Bad dreams?	0□No 1□< 1 time/week 2□1~2 times/week 3□ ⩾3 times/week
E13 Pain?	0□No 1□< 1 time/week 2□1~2 times/week 3□ ⩾3 times/week
E14 For other reasons that affect sleep, please explain	0□No 1□< 1 time/week 2□1~2 times/week 3□ ⩾3 times/week
E15 Your total sleep quality score for the last month: 0□Very good 1□Not bad 2□Not good 3□Too bad	
E16 Do you often have to take hypnotic pills (including buying them from a doctor's prescription or from an outside drugstore) before you can fall asleep?	0□No 1□< 1 time/week 2□1~2 times/week 3□ ⩾3 times/week
E17 Do you often feel sleepy?	0□No 1□< 1 time/week 2□1~2 times/week 3□ ⩾3 times/week

E18 Do you feel difficult and lack of energy when doing things?	0☐No 1☐Occasionally 2☐Sometimes 3☐Often

Table4

G1. Difficulty in falling asleep	None	Mild	Moderate	Heavy weight	Extremely heavy
	0	1	2	3	4
G2. Difficulty in sleep maintenance	None	Mild	Moderate	Heavy weight	Extremely heavy
	0	1	2	3	4
G3. Wake up early	None	Mild	Moderate	Heavy weight	Extremely heavy
	0	1	2	3	4
G4. How satisfied / dissatisfied are you with your current sleep patterns?	None	Mild	Moderate	Heavy weight	Extremely heavy
	0	1	2	3	4
G5. To what extent do you think your insomnia affects your daily function?	None	Mild	Moderate	Heavy weight	Extremely heavy
	0	1	2	3	4
G6. Your insomnia problem has affected your quality of life. What do you think of your insomnia in the eyes of others?	None	Mild	Moderate	Heavy weight	Extremely heavy
	0	1	2	3	4
G7. How worried / painful are you about your current sleep problems?	None	Mild	Moderate	Heavy weight	Extremely heavy
	0	1	2	3	4
Total score: _____					

Table5 NRS2002

1. Patient data

Name		Hospitalization number	
Gender		Inpatient Ward	
Age		Bed number	
Height(m)		Weight(kg)	
Body mass index (BMI)		Protein (g/L)	
Clinical diagnosis			

2.Disease Status

Disease Status	Score	Yes(√)
Patients with pelvic fractures or chronic diseases have the following diseases: liver cirrhosis, COPD, long-term hemodialysis, diabetes, tumor		
Major chest surgery, stroke, severe pneumonia, hematological tumor		
Craniocerebral injury, myelosuppression and intensive care patients (APACHE > 10)		
Total		

3.Nutrition Status

Nutritional status indicators (single selection)	Score	Yes(√)
Normal nutritional status		
5% weight loss in 3 months or 20% reduction in food intake (compared to requirements) in the last week.		
Weight loss in 2 months > 5% or BMI 18.5 ~ 20.5 or food intake in the last week (compared to requirement)		

reduced by 50% ~ 75%		
Weight loss in 1 month > 5% (or > 15% in 3 months) or BMI < 18.5 (or serum albumin < 35g/L) or food intake in the last week (compared to requirement) reduced by 70% ~ 100%		
Total		

4.Age

Over 70 years old plus 1 point		

5. Results of nutritional risk screening and assessment

Total score of nutritional risk screening	
