# Supplementary figures and images for: Sleep quality and influencing factors and correlation with T-lymphocyte subpopulation counts in patients with pulmonary tuberculosis: a cross-sectional study
Source: BMC Infect Dis. 2022 Dec 22;22:956. doi: 10.1186/s12879-022-07946-7 (PMC9773432; doi:10.1186/s12879-022-07946-7)

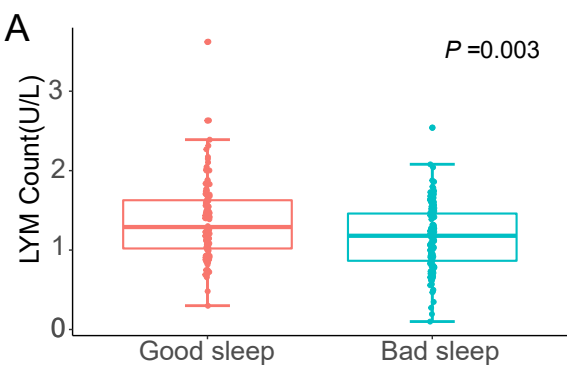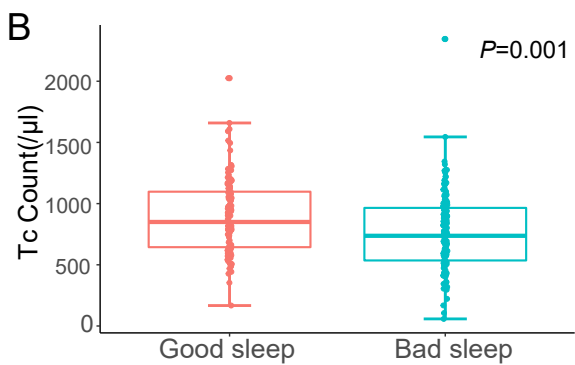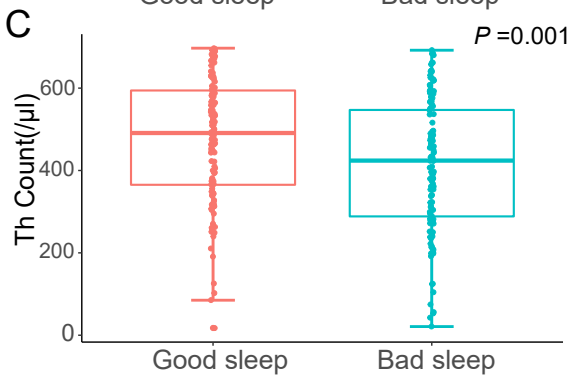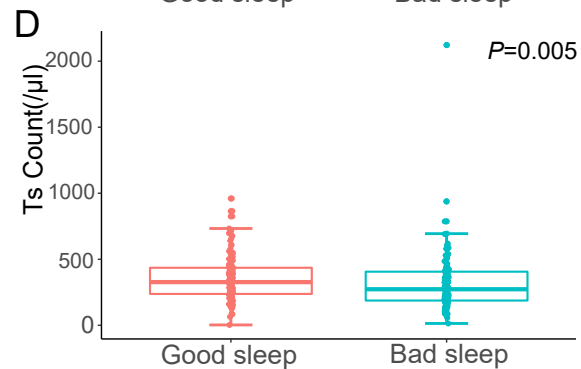

Supplement: Supplementary file 2 — Additional file 2. A subgroup analysis of immunological indicators was performed for the two groups of patients, and all variables with statistically significant differences were included in the figure. [file 12879_2022_7946_MOESM2_ESM.pdf]
